# Maternal allergic contact dermatitis causes increased asthma risk in offspring

**DOI:** 10.1186/1465-9921-8-56

**Published:** 2007-07-27

**Authors:** Robert H Lim, Mohamed S Arredouani, Alexey Fedulov, Lester Kobzik, Cedric Hubeau

**Affiliations:** 1 Department of Environmental Health, Harvard School of Public Health, Boston, MA, USA; 2 Department of Surgery, Beth-Israel Deaconess Medical Center/Harvard Medical School, Boston, MA, USA; 3 Department of Pathology, Brigham & Women's Hospital, Boston, MA, USA; 4 Department of Biology, Momenta Pharmaceuticals, Inc., Cambridge, MA, USA

## Abstract

**Background:**

Offspring of asthmatic mothers have increased risk of developing asthma, based on human epidemiologic data and experimental animal models. The objective of this study was to determine whether maternal allergy at non-pulmonary sites can increase asthma risk in offspring.

**Methods:**

BALB/c female mice received 2 topical applications of vehicle, dinitrochlorobenzene, or toluene diisocyanate before mating with untreated males. Dinitrochlorobenzene is a skin-sensitizer only and known to induce a Th1 response, while toluene diisocyanate is both a skin and respiratory sensitizer that causes a Th2 response. Both cause allergic contact dermatitis. Offspring underwent an intentionally suboptimal protocol of allergen sensitization and aerosol challenge, followed by evaluation of airway hyperresponsiveness, allergic airway inflammation, and cytokine production. Mothers were tested for allergic airway disease, evidence of dermatitis, cellularity of the draining lymph nodes, and systemic cytokine levels. The role of interleukin-4 was also explored using interleukin-4 deficient mice.

**Results:**

Offspring of toluene diisocyanate but not dinitrochlorobenzene-treated mothers developed an asthmatic phenotype following allergen sensitization and challenge, seen as increased Penh values, airway inflammation, bronchoalveolar lavage total cell counts and eosinophilia, and Th2 cytokine imbalance in the lung. Toluene diisocyanate treated interleukin-4 deficient mothers were able to transfer asthma risk to offspring. Mothers in both experimental groups developed allergic contact dermatitis, but not allergic airway disease.

**Conclusion:**

Maternal non-respiratory allergy (Th2-skewed dermatitis caused by toluene diisocyanate) can result in the maternal transmission of asthma risk in mice.

## Background

Asthma is a significant cause of morbidity and mortality [1] whose prevalence has almost doubled in the past 20 years [2]. The pathogenesis of asthma is multifactorial and not entirely understood. However, maternal asthma is a known risk factor for asthma in children [3-5]. There is also evidence in mice and humans that allergic sensitization may occur in the prenatal period [6-8]. This maternal association taken together with prenatal sensitization data, implies that some component(s) of the *in utero *environment may be causing increased asthma risk in offspring.

To investigate the mechanisms that mediate the maternal transfer of asthma risk, we developed a murine model in which offspring of asthmatic BALB/c female mice show higher asthma susceptibility than normal babies. Specifically, babies of ovalbumin (OVA)-sensitized and challenged mothers develop an asthma-like syndrome in response to an intentionally suboptimal protocol of allergen sensitization that has little effect on normal babies [9-11]. We have also recently reported that the adoptive transfer of allergen-specific T cells [12] is sufficient to recreate the maternal transfer of asthma risk, even though recipient mothers do not show detectable signs of allergic airway disease.

This finding suggested that the maternal effect occurs through systemic production of allergic mediators rather than asthma *per se*. We therefore postulated that various allergic stimuli could similarly increase the risk of asthma in offspring. To test this hypothesis we induced allergic contact dermatitis (ACD) in BALB/c female mice through topical applications of toluene diisocyanate (TDI) or dinitrochlorobenzene (DNCB), mated them, and assessed their offspring for asthma risk. Although both chemicals are potent skin sensitizers, TDI provokes Th2-dominated responses [13-16] and is also a known respiratory sensitizer implicated in occupational asthma after inhalation exposures [17]. Conversely, DNCB is considered a strict contact allergen that stimulates a Th1-type pattern of cytokine secretion [13,18] and has no reported effects on the airways [19]. Our data showed that maternal ACD mediated by TDI, but not by DNCB, results in increased asthma susceptibility in offspring. Since we previously found that IL-4 plays a prominent role in the maternal effect in OVA-allergic mothers [9], we also investigated its role by using IL-4 deficient mice in the ACD model.

## Methods

### Animals

Male and female BALB/c mice, 8–10 week-old were obtained commercially from Charles River Laboratories (Wilmington, MA). IL-4 knockout mice (BALB/c background) were obtained from Jackson Laboratories (BALB/c-*Il4*^*tm2Nnt*^/J, stock number 002496)[20]. Mice were housed and fed standard lab chow *ad libitum *in a pathogen-free barrier facility that was maintained at 22–24°C with a 12-h dark/light cycle. Animal experiments were conducted under a protocol approved by our institutional review board.

### TDI/DNCB-induced ACD

Our original model using ovalbumin (OVA)-sensitized and OVA aerosol-challenged mother mice was detailed elsewhere [9]. Normal BALB/c female mice received 2 topical applications on their backs (day 0 and 7) of either vehicle only (a mixture of acetone and mineral oil, 4:1, v:v), or 50 μl of 2% dinitrochlorobenzene (DNCB, Sigma-Aldrich, Saint Louis, MO), or 50 μl of 2% toluene diisocyanate (TDI, Sigma-Aldrich). The females were allowed to mate with normal BALB/c males 24 hours after the second chemical application (day 8). In some experiments, controls and treated mice received topical application of 1% hydrocortisone acetate (GC) on day 7 (4 hours after second chemical application) and day 8 (before mating) of the protocol. Additional GC applications were then performed every 4 days during the whole pregnancy.

### Allergen sensitization and challenge

**Figure 1:**
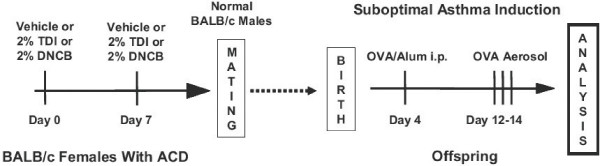
Schematic of main protocol. Vehicle, 2%TDI, or 2%DNCB was applied to wild-type female BALB/c mice on day 0 and 7, followed by mating on day 8. Offspring received one i.p. injection of OVA/Alum at day 4 of life, 10 minute aerosol challenges with 3% OVA on days 12–14, followed by analyses on days 15 and 16.

Offspring of treated/untreated mother mice were submitted to an intentionally suboptimal asthma induction protocol (see Fig. 1) that features only one single i.p. injection of 5 μg OVA in 1 mg Al(OH)3 on day 4 of life, followed by 3% OVA aerosols on days 12–14. Final physiologic and pathologic analyses were performed on days 15 and 16, respectively.

### Histology

Mother and baby mouse lungs were inflated with 10% buffered formalin, removed, and immersed in the same solution. In the case of skin samples, approximately half a square inch of the chemically-treated area was collected on day 15 of the protocol (1 week after second application) and immediately similarly fixed in 10% buffered formalin. All the tissue samples were embedded in paraffin and sections for microscopy were stained with hematoxylin and eosin (H&E).

### Cellularity in superficial cervical lymph nodes

The draining lymph nodes were collected from treated/untreated female mice 24 hours after topical application of DNCB and TDI. The 4 lymph nodes from each animal were rinsed in PBS and incubated for 1 hour (37°C) in 1 ml RPMI containing 10% fetal calf serum (FCS) and 1 mg collagenase D (Roche Diagnostics Corporation, Indianapolis, IN). The cells were then expelled through a 70-μm nylon mesh filter, washed in RPMI (10% FCS), and finally resuspended in PBS. Total cell counts per mouse (4 lymph nodes each) were determined by means of a hemocytometer.

### Pulmonary function testing and pathologic analysis

Airway bronchoconstriction in response to increasing concentration (0–100 mg/ml) of aerosolized methacholine (MCh) was evaluated in mothers and offspring 24 hours after last allergen exposure (chemicals or OVA) using unrestrained plethysmography (Buxco, Sharon, CT). The use of unrestrained plethysmography and enhanced pause (Penh) as surrogate of the mouse lung function has important limitations [21]. However, in BALB/c mice, from which the present results were exclusively obtained, enhanced pause (Penh) correlates well with invasive measures of lung resistance [22]. It is also worth noting that our models use cohorts of 2 week-old baby mice that are too small to be tested through invasive techniques and make unrestrained plethysmography a more appropriate technique [23]. Bronchoalveolar lavage (BAL) was performed on euthanized animals 48 hours after last allergen challenge (day 9 of the protocol for mothers, day 16 of life for offspring). Total cell yield was quantified by hemocytometer. BAL differential cell count was performed on cytocentrifuge slides stained with Diff-Quick (VWR, Boston, MA).

### Multiplex cytokine immunoassay

Serum samples were collected from each group of female mice at week 1, 2, and 3 of pregnancy and the offspring at time of euthanasia. Circulating cytokines were quantitated using multiplex biomarker immunoassays from Linco Research (St Charles, MI) and xMAP technology (Luminex, Austin, TX) according to the manufacturer's instructions. Standards and internal controls were added for each cytokine and diluted in serum-like medium. Raw data were analyzed using Masterplex QT 2.0 software (Luminex). Graphs are representative of data averaged from 3 to 5 animals per group, each sample assayed in duplicates. Final concentrations were expressed in pg/ml ± SEM. Concentrations plotted as 'zero' on the graphs were below detection limits.

### Statistical analysis

All data are presented as means ± SEM. Differences between groups were compared using one-way or two way ANOVA with protected least significant differences (PLSD) Fisher's test and Bonferroni post-tests. Prism GraphPad and StatView software program (Abacus Concepts, Berkeley, CA) were used for all statistical analysis. A p value < 0.05 was considered significant.

## Results

### Evaluation of maternal skin and respiratory responses

**Figure 2:**
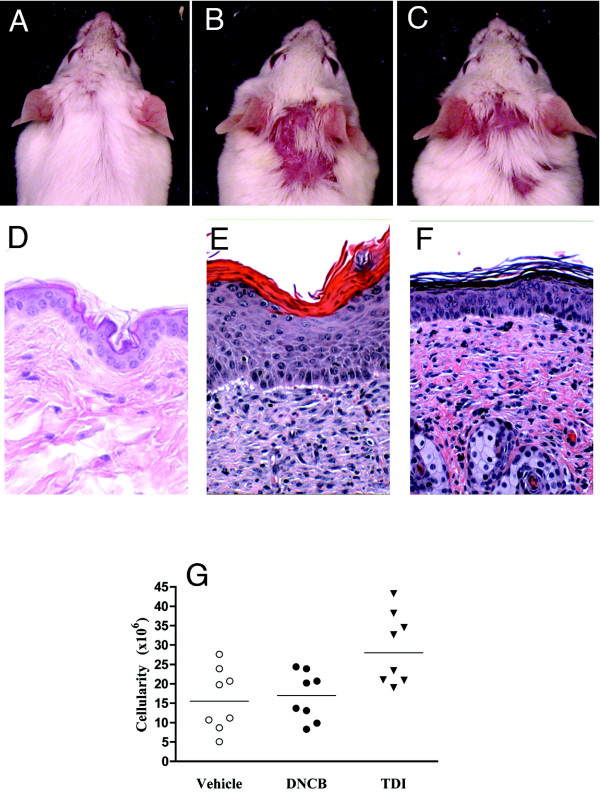
Effect of vehicle, DNCB, and TDI on adult female mice. 2A-C shows gross appearance of skin after 2^nd ^application of irritant (vehicle, DNCB, TDI; respectively). 2D-F shows inflammation of dermal layers in DNCB (2E) and TDI (2F) treated mice compared to vehicle (2D) treated mice. 2G shows increased lymph node cellularity in TDI group compared to others (*p < 0.005).

**Figure 3:**
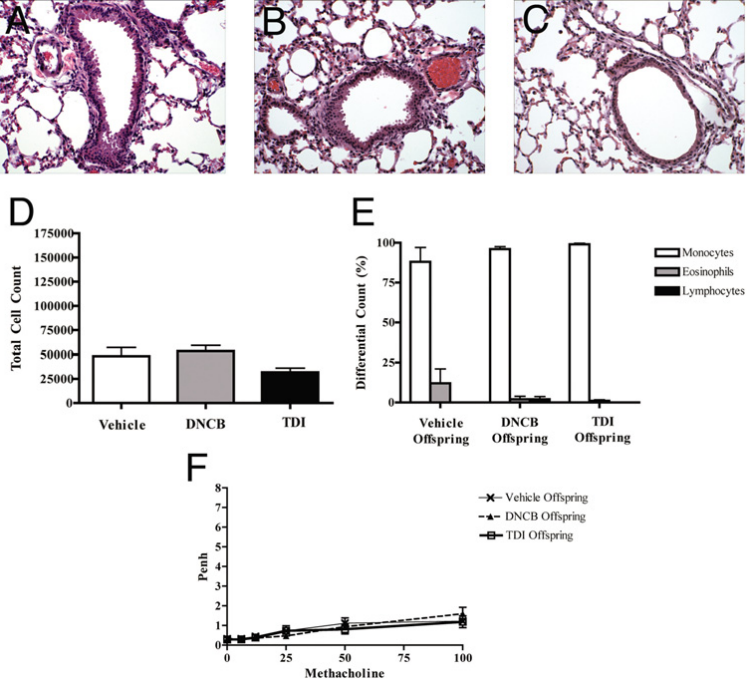
Effect of vehicle, DNCB, and TDI on airways of adult female mice. 3A-C shows normal histology of airways of mothers treated with DNCB (B) and TDI (C), similar to vehicle (A). 3D and E show that all groups had similar BAL total cell counts and percentage eosinophils. 3F shows similar minimal responsiveness to methacholine as measured by Penh in all groups.

After both DNCB and TDI application, macroscopic changes including hair loss and local erythema were seen (Fig. 2B and 2C, respectively). Histopathology of the skin at the area of chemical irritant application showed increased dermal thickness (Fig. 2E – DNCB and Fig. 2F – TDI) and inflammation. While the local skin lesions were similar, only the TDI treated group showed showed increased cellularity in draining lymph nodes, as compared to vehicle- and DNCB-treated mothers (Fig. 2G). Although TDI is a known respiratory sensitizer, it is not known to cause airway disease after exposures limited to topical skin contact. We performed a series of analyses to more directly determine the effects, if any, on the airways of mothers treated topically in our protocols. Histopathologic evaluation of maternal lungs showed normal structure (Fig. 3A – Vehicle, 3B – DNCB, and 3C – TDI). BAL total cell and differential counts were similar in mothers across all treatment groups (Fig. 3D and 3E). Airway responsiveness was similarly minimal in all groups (Fig. 3F). The serum cytokine levels in all groups showed statistically equal and low amounts of GM-CSF, IL13, IFN-g, and IL-5 (p values > 0.05). IL-4 levels were below the limits of detection in all groups (data not shown). These data indicate that the mothers in all treatment groups did not have signs of allergic respiratory disease.

### Increased Asthma Susceptibility in Offspring of TDI treated mothers

**Figure 4:**
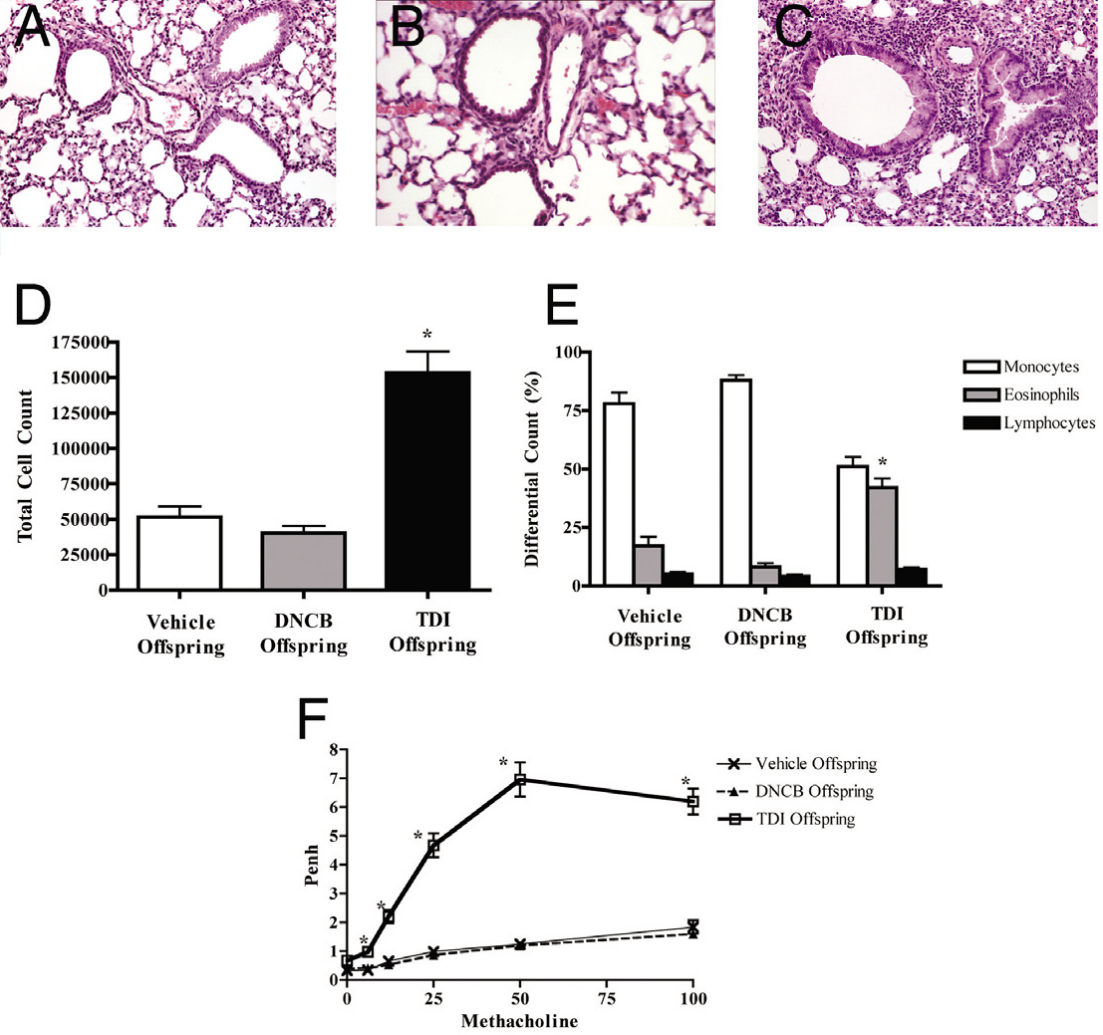
Maternal ACD and offspring susceptibility to OVA-induced asthma. Following the suboptimal protocol, an increase in mononuclear and eosinophilic cells is seen in airways of offspring from mothers treated with TDI (C), versus DNCB (B) and vehicle-treated mothers (A). Total cell counts and eosinophil percentages are higher in TDI offspring (4D-E, *p < 0.05). Penh values were elevated in TDI offspring (4F, *p < 0.05).

**Figure 5:**
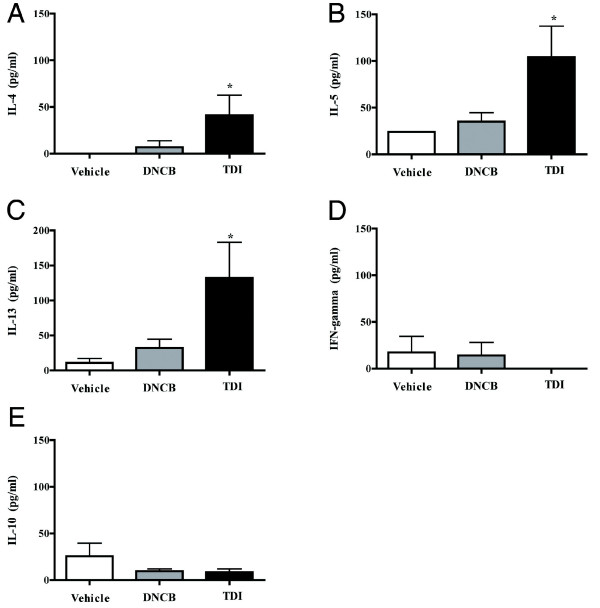
BAL cytokine levels in offspring of mothers treated with vehicle, TDI, and DNCB. TDI offspring had significantly higher levels of IL-4 (A), IL-5 (B), and IL-13 (C) (*p < 0.05) as compared to offspring of DNCB and vehicle treated mothers. IFN-g (D) and IL-10 (E) levels were similar in all groups.

Female mice were treated topically twice with vehicle, DNCB, and TDI, mated, and their offspring were subjected to the protocol summarized in Fig. 1. Histologic evaluation showed markedly increased inflammatory infiltration in the lung samples of the offspring of TDI-treated mothers (TDI offspring, Fig 4C) compared to offspring of DNCB- and vehicle-treated mothers (DNCB offspring and vehicle offspring; Fig 4B and 4A, respectively). This infiltrate was primarily found around airways and vessels, and composed of mononuclear cells and eosinophils. Quantitative analysis of bronchoalveolar lavage confirmed the qualitative histologic findings. We found significantly increased total cell counts in the TDI offspring, as compared to the DNCB and vehicle offspring (Fig. 4D, p < 0.05). Further, the TDI offspring showed significantly increased eosinophilia (Fig. 4E, p < 0.05). Airway responsiveness, as measured using unrestrained plethysmography, was also increased in the TDI offspring at all methacholine concentrations as compared to DNCB offspring and vehicle offspring (Fig. 4F, p < 0.05). The cytokine profile in the BAL of the TDI offspring showed Th2 skewing, with significantly increased IL-4, IL-5, and IL-13 (Fig. 5, p < 0.05). IFN-g and IL-10 showed a trend toward decrease in the TDI offspring as compared to vehicle and DNCB offspring, although this did not achieve statistical significance.

### Topical treatment with glucocorticoids after development of ACD from TDI exposure

We sought to test the postulate that therapeutic modulation of the TDI-mediated ACD would reduce the increased asthma susceptibility observed in the TDI offspring. To this end, after induction of ACD with TDI or DNCB, glucocorticoids (TDI-GC or DNCB-GC) were applied to the skin lesions on 2 separate occasions prior to mating. We found no effect of the GC therapy. For the TDI and TDI-GC groups, the BAL total cell counts (140,000 ± 20,745 and 175,833 ± 16,235, respectively) and percentage eosinophilia (48% ± 7.34 and 56% ± 4.76, respectively) were statistically similar (p > 0.05). The Penh values were also similar between these two groups (p > 0.05). Both the TDI and TDI-GC group showed statistically increased total cell count, percentage eosinophilia, and Penh compared to negative controls. For the DNCB and DNCB-GC groups, BAL total cell counts, percentage eosinophilia, Penh were statistically similar (p > 0.05) and no different then negative controls (p > 0.05). Also after application of the GC, no visible change in the ACD lesions was noted.

### IL-4KO mothers transfer asthma susceptibility

In a prior study, injections of IL-4 monoclonal antibody (mab) in OVA-sensitized and challenged mothers attenuated the maternal effect, implying that this cytokine played a significant mechanistic role in the maternal transfer of asthma risk. To examine the role of IL-4 in the maternal transfer seen using the ACD model, we performed experiments using IL-4 KO females. After induction of ACD, IL-4 KO females were mated with normal males, and their heterozygote offspring tested in the protocol summarized in Fig. 1.

**Figure 6:**
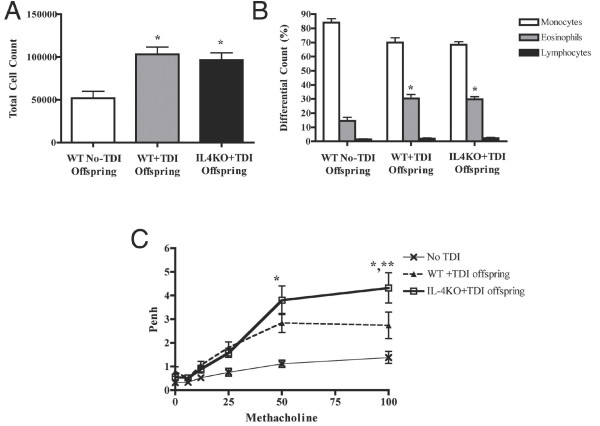
IL-4 and ACD-mediated maternal transmission of asthma risk. BAL total cell counts and percentage eosinophils in IL4KO+TDI and WT+TDI offspring were elevated compared to WT NO-TDI offspring (Fig A and B, *p < 0.05). Penh values for IL4KO+TDI and WT+TDI offspring were significantly greater than WT NO-TDI offspring (Fig C, *p < 0.05). Penh for IL4KO+TDI and WT+TDI offspring were statistically similar, except at 100 mg/ml methacholine (**p < 0.05).

The offspring of wild type mothers treated with TDI (WT+TDI offspring) and the offspring of IL-4KO mothers treated with TDI (IL-4KO+TDI offspring) both had significantly increased BAL cell counts as compared to offspring of untreated mothers (WT NO-TDI offspring) (Figure 6A). Cell counts were not significantly different between WT+TDI offspring and IL-4KO+TDI offspring. Differential counts showed statistically increased eosinophilia in both the WT+TDI offspring and IL-4KO+TDI offspring as compared to negative control offspring (Fig. 6B, p < 0.05). Penh values for the TDI treated groups (WT+TDI offspring and IL-4KO+TDI offspring) were also both significantly higher as compared to negative controls (Fig. 6C, p < 0.05). The increased Penh values for the IL-4KO+TDI offspring and WT+TDI offspring were similar, except for slightly greater Penh in the IL-4KO group at the highest methacholine concentration.

## Discussion

The goal of these experiments was to determine if non-respiratory allergy can result in the maternal transmission of asthma risk. Expanding on a previously described mouse model, we recently observed that maternal asthma *per se *was not needed to transfer asthma risk. The mothers needed only to be injected with DO11.10 T-cells (which bear an OVA-specific T cell receptor) and receive OVA challenge prior to mating to increase the asthma susceptibility in offspring (16). Based on these data, we postulated that any maternal Th2-skewed allergic state might similarly increase asthma susceptibility in offspring. In the current study we tested this hypothesis by inducing ACD in mothers using Th1 or Th2 inducing skin irritants (DNCB or TDI, respectively) and assessing the offspring for susceptibility to develop an asthmatic phenotype. The offspring of mothers with DNCB-ACD and those treated with vehicle alone did not have increased susceptibility, while the offspring of the TDI-ACD mothers did show allergic airway inflammation and increased Penh responses to methacholine. Moreover, the cytokine profile of the offspring for the TDI group was Th2 skewed as compared to DNCB and control groups. Hence by multiple criteria, these pups exhibited an asthma-like phenotype after the intentionally suboptimal protocol used in our models (Fig 1). Although both the DNCB and TDI treated groups had significant and similar degrees of ACD, it is noteworthy that the mothers' airways showed findings essentially identical to controls, with no evidence of pulmonary inflammation. Thus, the data indicate that transfer of asthma susceptibility can follow a maternal allergic (Th2) response in another tissue site without a requirement for pulmonary allergy.

As maternal ACD caused increased susceptibility to asthma in offspring, we postulated that modulation of the ACD might affect transmission of asthma risk. However, treatment of the lesions with topical glucocorticoids did not affect the offspring's response to the suboptimal protocol. Since no gross difference was seen in the ACD lesions between GC-treated and untreated groups, the dose used may have been insufficient. Another possibility is the timing of treatment (after the second topical application) missed an earlier therapeutic window.

Because in a prior study, the maternal effect could be attenuated with a single injection of IL-4 mab, we postulated that IL-4 could also play a pivotal role in the maternal transmission of asthma in the TDI ACD group. We predicted that mothers who lacked IL-4 production would have decreased ability to transmit asthma risk to offspring. Instead, the offspring of IL-4KO mothers treated with TDI had increased asthma susceptibility similar to that seen in offspring of wild-type mothers. This argues against IL-4 alone as a required mediator of the TDI-induced maternal effect. This may indicate that in the original study, the IL-4 mab was crossing the placenta and acting directly on the fetus, rather then acting primarily on the mother, though that seems unlikely based on the design of that study (injection pre-mating). More likely, this finding indicates differences in the mechanisms of maternal transmission of asthma risk in the two models. A study by Matheson et al [14] showed that their model of TDI induced asthma required depletion of both IL-4 and IL-13 in order to ablate the asthmatic phenotype. It may be that deficiency in both these Th2 cytokines is required for abrogation of the maternal effect in a TDI-ACD model, a postulate that may be amenable to direct testing in future studies.

There are some limitations of this study that merit discussion. In experiments using IL-4KO, there were statistically significant differences in Penh, cell counts and differentials. However, the absolute values were decreased as compared to other experiments using wild-type mice. Because duplicate experiments showed similar results, we consider the results valid for interpretation as not supporting a critical role for IL-4 in the TDI-ACD group. A second limitation is the use of unrestrained plethymography, whose use has become controversial. However, in BALB/c mice, from which the present results were exclusively obtained, Penh has proven to highly correlate with lung resistance [22]. Further, the Penh measurements were correlated with cell counts, differentials, and histopathology. Also, more invasive means of measuring airway dynamics are not possible in the 2 week old animals studied in our protocol. A third issue in this study relates to the increase in lymph node cellularity seen in the TDI versus DNCB treated mothers. One previous study by Lee *et al *[24] also examined the cellularity of draining lymph nodes following TDI and DNCB exposure at various concentrations. They found that, at higher concentration of DNCB (0.5%–1%) and at all concentrations of TDI, treated mice had increased cellularity as compared to vehicle. However, the TDI treatment group did appear to have increased cellularity as compared to DNCB. This apparent disagreement with our data may be due to a difference in technique. Their mice received the irritants on 3 consecutive days, rather then twice spaced apart by 7 days. However the importance of this apparent disagreement is difficult to assess as it is not clear that the differences in the cited study were statistically significant.

The data obtained in this study support the conclusion that a Th2 dominated-allergic response, regardless of tissue site, is sufficient for the maternal transmission of asthma risk. Although this study does not resolve the mechanisms involved, it does allow exclusion of some possibilities and raises some interesting issues. Based on this and previous studies, specific maternal antibodies or immunoglobulins are not required to transfer risk, as increased susceptibility to OVA-allergy was seen in offspring of mothers who were OVA-naive. Our study also rules out a requisite role for IL-4 in the TDI-ACD maternal effect, as IL-4KO mothers were able transmit asthma susceptibility to their offspring. We did not find evidence that maternal serum cytokine levels impact maternal transfer of asthma risk, as in all groups, the serum levels of multiple Th1 and Th2 cytokines were not significantly different, although an exhaustive analysis of multiple cytokines and timepoints was not done, precluding definitive interpretation of these negative results.

The only difference observed between the vehicle, DNCB, and TDI mothers was the cellularity of their lymph nodes. We speculate that important clues for the mechanisms for maternal transmission of asthma may be found by analysis of the cell types expanded in the nodes. A specific issue to be addressed is raised by the recent finding that DNCB may induce a mixed Th1/Th2 response under certain circumstances. In a recent study by Vanoirbeek *et al *[25], it was shown that mice sensitized and then exposed to DNCB had increases in Con A-stimulated IL-4 release by cultured lymph node lymphocytes. Evaluation of the lymph node populations and function (e.g., cytokine secretion) has proven useful in numerous previous studies focused on ACD pathogenesis. For example, ACD is known to cause migration of Langerhans cells (LC) and dermal dendritic cells (DC) to draining lymph nodes, after they have encountered and internalized antigen found in the skin. Once at the draining nodes, they cause expansion of a receptive sub-population of T-lymphocytes [26]. It is likely that the ACD caused by the Th2 skewing contact irritant causes increased migration of LC and DC to the draining nodes, which cause a clonal expansion of a subset of T-cells. This subset may then be responsible for the maternal effect. Prior studies have shown that CD4+T-cells are essential for the development of isocyanate induced asthma [14,27], and the latter study showed that CD8+T-cells are important for the induction of dermatitis. One or both of these subsets may be the T-cell subset seen in the draining lymph nodes of the TDI treated mother mice. The TDI-ACD model will allow further analysis of whether and how these subsets play a pivotal role in the maternal transmission of asthma risk.

## Abbreviations

AHR, airway hyperresponsiveness; Penh, enhanced pause; TDI, toluene diisocyanate; DNCB, dinitrochlorobenzene; BAL; bronchoalveolar lavage.

## Competing interests

The author(s) declare that they have no competing interests.

## Authors' contributions

RL carried out the unrestrained plethysmography, BAL, injections, nebulizations, skin irritant treatments, and drafted the manuscript. MA performed the lymphnode extraction and analysis. AF performed some of the nebulizations. LK conceived the study and helped to draft the final manuscript. CH helped with the conception of the study, as well as the unrestrained plethysmography, BAL, injections, nebulizations, multiplex cytokine assays, and skin irritant treatments. All authors read and approved the final manuscript.
